# ROS and Endoplasmic Reticulum Stress in Pulmonary Disease

**DOI:** 10.3389/fphar.2022.879204

**Published:** 2022-04-26

**Authors:** Xiangning Cui, Yang Zhang, Yingdong Lu, Mi Xiang

**Affiliations:** ^1^ Department of Cardiovascular, Guang’anmen Hospital, China Academy of Chinese Medical Sciences, Beijing, China; ^2^ First Clinical Medical School, Shandong University of Traditional Chinese Medicine, Jinan, China

**Keywords:** er stress, unfolded protein response, pulmonary disease, reactive oxygen spieces, oxidative stress

## Abstract

Pulmonary diseases are main causes of morbidity and mortality worldwide. Current studies show that though specific pulmonary diseases and correlative lung-metabolic deviance own unique pathophysiology and clinical manifestations, they always tend to exhibit common characteristics including reactive oxygen species (ROS) signaling and disruptions of proteostasis bringing about accumulation of unfolded or misfolded proteins in the endoplasmic reticulum (ER). ER is generated by the unfolded protein response. When the adaptive unfolded protein response (UPR) fails to preserve ER homeostasis, a maladaptive or terminal UPR is engaged, leading to the disruption of ER integrity and to apoptosis, which is called ER stress. The ER stress mainly includes the accumulation of misfolded and unfolded proteins in lumen and the disorder of Ca^2+^ balance. ROS mediates several critical aspects of the ER stress response. We summarize the latest advances in of the UPR and ER stress in the pathogenesis of pulmonary disease and discuss potential therapeutic strategies aimed at restoring ER proteostasis in pulmonary disease.

## 1 Introduction

The endoplasmic reticulum (ER) is a membrane organelle widely found. In eukaryotic cells with multiple functions. The ER coordinates secretory and transmembrane proteins folding and translocation, serves as a place to regulate the cellular stress response (Chen and Cubillos-Ruiz, 2021). The ER controls cellular Ca^2+^ uptake, storage and signaling, keeping the intracellular Ca^2+^ homeostasis. Meanwhile, the ER takes part in the generation of phospholipids including cholesterol (Sozen and Ozer, 2017). The ER lumen is the main site where proteins synthesize in cells. The accumulation of some misfold or unfolded proteins within the ER or the disruption of Ca^2+^ homeostasis can lead to the disequilibrium of ER homeostasis, which is called ER stress (Di Conza and Ho, 2020). The stability of the environment within the ER is the guarantee of regulating its normal function. In the early stage of ER stress, the induction of unfolded protein response (UPR) can reduce protein synthesis and enhance the degradation function of ER, thus lowering the burden of ER and maintaining intracellular homeostasis, which is a pro-survival response. With the aggravation of ER, the activation of UPR is inhibited, resulting in Ca^2+^ uptake/release barrier of ER or protein processing/transporting obstacle (Song et al., 2018). The signaling is accordingly transformed from survival to apoptosis, and the expression of ER stress-specific pro-apoptotic transcription factor CHOP is activated, further inhibiting the activity of AKT, and promoting cell apoptosis (Sen, 2019). The normal physiological function of ER is closely related to the redox state, and increasing studies suggest the production of ROS has become a primary part of ER stress response, possibly acting as an upstream signaling (Zhang et al., 2019a). The lung is in direct contact with the outside world, and external stimuli can easily lead to mis-synthetic proteins and cause the occurrence of pulmonary diseases including pulmonary fibrosis, pulmonary infections, asthma, COPD (chronic obstructive pulmonary disease) and lung cancer (Liu et al., 2017; Katzen and Beers, 2020). Several pathophysiological factors such as hypoxia, inflammation, and metabolic disorders can trigger ER stress. Conversely, ER stress induces inflammation and oxidative stress, maladaptive UPR induces apoptosis, and disruption of interaction between the ER and other organelles in epithelial cells can negatively affect pulmonary disease. In this context, we introduced the relationship between ROS and ER stress, summarized current research on the molecular mechanisms of ER stress in pulmonary disease, and raised several promising therapeutic strategies targeting ER stress that might prevent or treat pulmonary disease.

## 2 The Physiological Mechanism of Reactive Oxygen Species

ROS are oxygen radicals in organisms, including oxygen and oxygen-containing highly reactive molecules (such as superoxide anions, tissue peroxides, and free radicals), serving as an intracellular and inter-cellular messenger which regulates many signaling molecules (Incalza et al., 2018). ROS are generated as a byproduct in cells by mitochondria and other cellular organelles (Boengler et al., 2017). ROS modulate different cell signaling pathways, which are mostly mediated by these transcription factors NF-κB and STAT3, hypoxia-inducible factor-1*α*, kinases, cytokines, and other proteins; these signaling pathways have been demonstrated to be related with inflammation, proliferation, cellular transformation, angiogenesis, as well as cell metabolism in cancer cells (Prasad et al., 2017). Continuous studies have confirmed ROS production in hypoxia and ischemia models, and found that ER proteins were oxidized and modified, suggesting that ER functions can be destroyed by ROS (Xu et al., 2017; Wang et al., 2018a; Singh-Mallah et al., 2019). ROS production on ER is generated by delivering electrons to O^2^ by NADH-cytochrome P450 reductase to form O^2−^, with electrons delivered to O^2^ by the electron transport chain on the nuclear membrane, assisted by NADH (Delaunay-Moisan and Appenzeller-Herzog, 2015; Zeeshan et al., 2016). Changes of intracellular redox state promotes the production of oxygen radicals and apoptosis inducer activation, resulting in apoptosis and aggravate intracellular redox state variation, then affect cell physiological state, initiate oxidative stress, and trigger the activation of apoptotic signal activation. Furtherly, calcium imbalance, oxidative stress are also important factors to induce ER stress (Kaminskyy and Zhivotovsky, 2014; Prasad et al., 2017).

## 3 Endoplasmic Reticulum Function and Homeostasis

The ER is a multifunctional organelle that forms the access into the secretory pathway which is made up of smooth and rough endoplasmic reticulum. The ER is crucial in maintaining cellular calcium homeostasis, lipid biosynthesis and secretory, and transmembrane protein folding and translocation (Rowland and Voeltz, 2012; Schwarz and Blower, 2016).The oxidative environment of ER lumen promotes disulfide bond formation, allowing complex secretion and transmembrane protein folding. The process of protein folding is regulated by a variety of chaperones, folding enzymes and cofactors (Kim et al., 2013a; Saibil, 2013). The ER takes part in lipids production in cells such as cholesterol and glycerophospholipids regulated by multiple ER-localized lipid biosynthetic enzymes including GTPase SAR1B and SURF4 (Wang et al., 2021). When cholesterol levels in the ER turn too high, SREBP undergo conformational changes that hinder it from leaving the ER. Correspondingly, homeostasis of cholesterol is restored (Brown et al., 2018; Lim et al., 2019). The ER also participates in mediating calcium homeostasis in cells. In quiescent cells, the level of free Ca^2+^ in cytoplasm was lower than that in extracellular space and ER lumen. Disruption of intracellular Ca^2+^ homeostasis is related with inflammatory responses. Research suggest that increase in IP3R (inositol 1,4,5-trisphosphate receptor) mediated Ca^2+^ release can cause inflammatory pathophysiology of ventilator-induced lung injury in mice models *via* ER stress (Ye et al., 2021). During chaperone-assisted disulfide bond formation between polypeptide chain substrates, two electrons are provided to the cysteine residue within the PDI active site. This transfer of electrons results in the reduction of the PDI active site and oxidation of the substrate, suggesting main ER-originating ROS production process. Studies have confirmed that the ER membrane-associated protein ERO1 (Endoplasmic reticulum oxidoreductin-1) oxidizes PDI, indicating that ERO1 is closely associated with protein load in the ER and can trigger ROS generation and contribute to ER stress. In addition, experimental analysis revealed that UP to 25% of ROS is produced by disulfide bonds in the endoplasmic reticulum during mass oxidation. Therefore, the close relationship between ROS and endoplasmic reticulum homeostasis is further suggested.

## 4 Reactive Oxygen Species and Endoplasmic Reticulum Stress

As is illustrated above, accumulation of some misfolded or unfolded proteins within the ER or disruption of Ca^2+^ homeostasis can cause ER stress. Under normal circumstance, the stability of the environment within ER is the guarantee for the normal function of ER. In the early stage, ER stress reduces protein biosynthesis and enhances ER degradation function by inducing unfolded protein response (UPR), thus reducing ER burden, and maintaining intracellular homeostasis, which in essence is a protective means. Changes of intracellular redox state initiate oxidative stress and trigger the activation of apoptosis signals. Meanwhile, Ca^2+^ imbalance and oxidative stress are also important factors to induce ER stress.

### 4.1 Reactive Oxygen Species and UPR

#### 4.1.1 Unfolded Protein Reaction and its Relevant Signaling Pathways (Figure 1)

ER is an important site for secretory protein folding and various post-translational modifications such as glycosylation and disulfide bond formation. The homeostasis regulation of ER ensures that correctly folded proteins proceed to the next step of modification, while slowly folded or unfolded proteins are retained in the ER for proteasome degradation through ER related protein degradation, which is the unfolded protein response (UPR).

**FIGURE 1:**
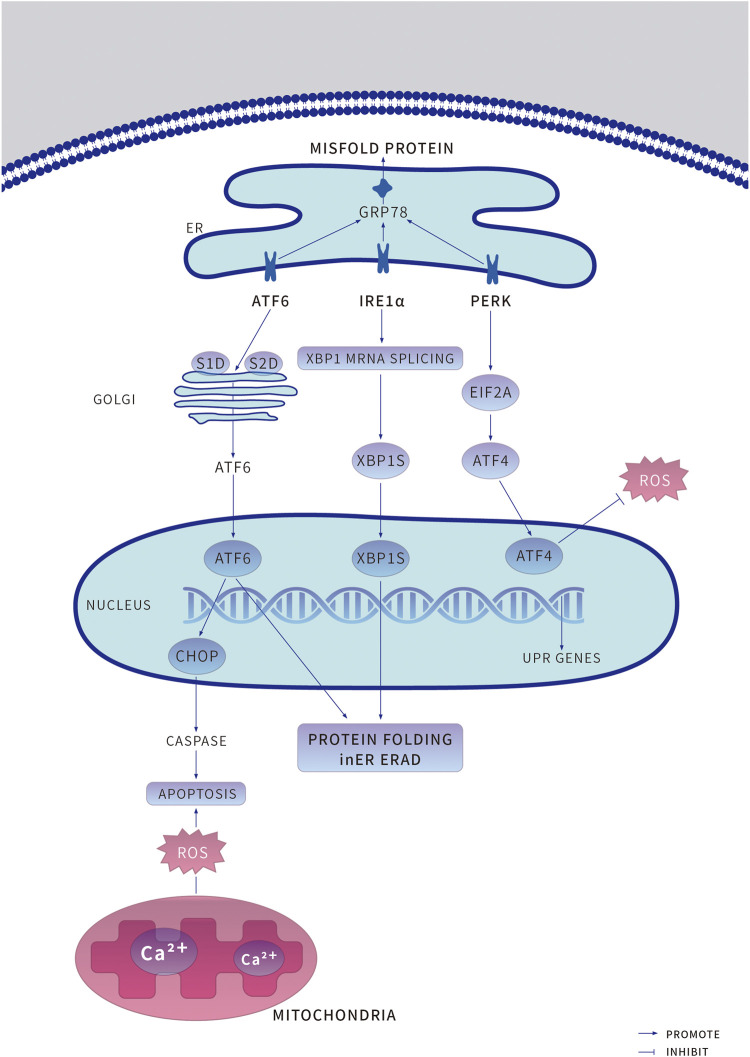
Under endoplasmic reticulum (ER) stress, the adaptive unfolded protein response (UPR) is induced to preserve protein homeostasis and to promote cell survival. An increased accumulation of unfolded or misfolded proteins causes the ER chaperone binding immunoglobulin protein (GRP78) to bind to misfolded proteins accumulated in the ER, resulting in the release of the UPR sensors protein kinase RNA-like ER kinase (PERK), Transcriptional transcription factor 6 (ATF6) and inositol-requiring protein 1*α* (IRE1*α*) and initiation of the respective proteins dimerization and phosphorylation of PERK and IRE1*α* activate their downstream pathways, PERK -- phosphorylated Eukaryotic Initiation factor 2*α*. Eukaryotic initiation factor 2A (EIF2A) -- ATF4 and IRE1*α* -- Spliced X-box-binding protein 1 (XBP1s), to promote cell survival. ATF6 is recruited to the Golgi apparatus for processing by the enzymes site-1 proteases (S1P) and site-1 proteases (S2P) to release cleaved ATF6, which enters the nucleus to induce the expression of target genes to promote cell survival. ERAD, ER-associated degradation; ROS, reactive oxygen species.

Under physiological conditions, three independent branches regulate the UPR, mediated by ER stress receptor proteins, PERK (RNA-dependent protein kinase (PKR)-like ER kinase), ATF6 (activating transcription factor 6), and IRE1*α* (Inositol-requiring enzyme-1*α*). These three individual proteins respectively bind to ER chaperone GRP78 (glucose-regulated protein 78) and remain inactive and serve as sensors and effectors for enhanced ER stress (Oakes and Papa, 2015; Kopp et al., 2019). When the nucleotide-binding domain of GRP78 coordinates with luminal domains of IRE1*α* and PERK, GRP78 is converted from a molecular chaperone to an ER stress sensor, accelerating further UPR activation (Kopp et al., 2019). These mechanisms can monitor the deviant conditions on the ER lumen, deliver signals to the cytosol, then transfer them the nucleus and activate corresponding downstream responses (Figure 1) (Schwarz and Blower, 2016).

PERK, a crucial ER stress sensor of the UPR, is particularly enriched at the MAMs (mitochondria-associated ER membranes), and capable of preventing mRNA translation under ER stress, thus inhibiting protein synthesis, and folding (Verfaillie et al., 2012). ATF4 (activating transcription factor 4) is a stress-induced transcription factor which is at always upregulated in cells, controls the expression of several adaptive genes that protect cells from stress, such as hypoxia or amino acid limitation. But under sustained stress conditions, ATF4 can initiate the transcription of CHOP (C/EBP homologous protein) to induce cell apoptosis (Wortel et al., 2017). PERK phosphorylates eIF2*α* (eukaryotic initiation factor 2) to decrease the GTP-bound (guanosine triphosphate, GTP) form and allow translation of ATF4 (Han et al., 2013; Luhr et al., 2019; Vanhoutte et al., 2021). Meanwhile, CHOP can bind to BOK (B-cell lymphoma 2 varian killer) and Caspase to act as an antiapoptotic agent (Carpio et al., 2015).

ATF6 is a type II transmembrane protein that induces ER stress response. As an up-regulating chaperone, it is an important element of the ERAD (endoplasmic reticulum—associated degradation) pathway (Jin et al., 2017). The unfolded proteins in the ER lumen separate ATF6 from GRP78, then ATF6 is transferred from the ER membrane to the Golgi apparatus as vesicles; the lumen domain of ATF6 is cleaved by S1P (serine protease site-1), the n-terminal is cleaved by metalloproteinase S2P (site-2 protease), forming the ATF6f with n-terminal cytoplasmic domain. As a result, several ER stress response element genes such as GRP78, GRP94, CHOP can be activated, and promote the ER associated protein degradation gene XBP1 (X-box binding protein 1) transcription. The ATF6f can bind XBP1 and control the expression of some specific genes (Zhang et al., 2019b; Pachikov et al., 2021).

IRE1*α* is a transmembrane protein, which is composed of n-terminal ER lumen sensor domain, a single transmembrane domain, and c-terminal cytoplasmic protein kinase effector, responsible for protein kinase and endonuclease activity. When ER stress happens, IRE1*α* cavity structure domain is dissociative with GRP78, causing cavity structure domain two polymerization to further activate the activity of enzymes, catalyze the transcription of XBP1 to upregulate multiple folding enzymes, including glycosylation, redox enzymes degradation to rectify ER homeostasis. IRE1*α* also degrades specific mRNA in a tissue-specific manner through RIDD (regulated IRE1-dependent mRNA decay) and activates related kinases through binding of adaptor proteins including JNK (C-Jun amino-terminal kinase) and ASK1 (apoptotic signal-regulated kinase 1) pathways (Lerner et al., 2012; Hwang and Qi, 2018; Huang et al., 2019).

Adaptive UPR is the initial defense against ER stress to keep the balance of ER homeostasis. However, persistent UPR activation can transform into maladaptive UPR, leading to drastic cell injuries even cell death. Incorrectly or terminally misfolded proteins are retained and degraded within the ER. As ER stress cells evolve further with the integrated stress response, cells actively mobilize stress response proteins to resist the deleterious effects caused by stress triggers. Meanwhile, cells adjust ER function to adapt to new internal environmental change requirements. On the other hand, some stress protein regulatory genes that may cause cell death, such as CHOP, will decide according to the situation to finally clear those ER stress cells that are simply unable to return to a normal functional state (Shirakawa et al., 2013; Hotamisligil and Davis, 2016; Urra et al., 2016; Hetz and Papa, 2018). In ER stress response, the activation of IRE-1, PERK, and ATF6 induces CHOP, promotes the activation of CHOP, and significantly increases its expression, leading to apoptosis. IRE-1-mediated splicing of XBP1 induced UPR can promote cell survival, but its overexpression can also promote cell apoptosis. Activated IRE-1 can combine with TRAF2 (TNF-receptor-associated factor 2) and ASK1 (apoptotic signal-regulated kinase 1) to form into the IRE-1-TRAF2-ASK1 complex on ER outer membrane, which activates JNK and P38 MAP (mitogen-activated protein kinase) (Matsuzawa et al., 2002). CHOP expression is induced by phosphorylation of ser78/81 in the transcriptional activation domain of P38 MAP kinase. After ER stress induced GRP78 dissociation from ATF6, the cytoplasmic ATF6 n-terminal domain also dissociated from ER membrane and formed a 50 ku active fragment. It was found that the activated ATF6 entered the nucleus and bound to the NF-Y (nuclear transcription factor-Y) and bound to the promoter ER stress element with homologous or heterologous dimer structure. The complex then induces the transcriptional expression of ER stress genes including CHOP (Feng et al., 2019). PERK is also a transmembrane protein. After dissociation from GRP78, PERK is activated by self-dimerization and phosphorylation of the intracellular domain. Activated PERK phosphorylates serine of the eukaryotic translation initiation factor eIF2*α*. Phosphorylated eIF2*α* cannot accept the exchange of GTP-GDP by eIF*β*, thus slowing or suspending protein synthesis, thereby promoting the activation of CHOP, and thus inducing cell metabolism and apoptosis (Pillai, 2005; Kopp et al., 2019).

#### 4.1.2 Reactive Oxygen Species Induced Unfolded Protein Reaction

ROS can directly attack the free thionyl group, necessary to maintain the protein foldase activity, leading to oxidative modification of proteins in the ER lumen, and then induce abnormal function of ER molecular chaperones, resulting in the accumulation of unfold proteins and retention in the ER lumen, ultimately triggering ER stress. At the same time, the expression of the chaperone protein GRP78 significantly increased and dissociated from the three transmembrane proteins to bind unfolded proteins to enhance ER protein folding ability and reduce protein accumulation in ER. After dissociation of GRP78, ER stress receptor proteins are activated to trigger UPR generation, reduce the load of ER by limiting the synthesis of unfold proteins, and trigger their respective induced pro-survival response. The PERK-activated-pro-survival pathway inhibits protein translation and synthesis by limiting the entry of abnormally folded proteins into ER and reducing ER’s pressure for new protein folding (Borges and Lake, 2014; Hetz et al., 2015; Khanna et al., 2021). PDI (phosphorylation of protein disulfide isomerases), fundamental enzymes involved in ER protein folding, allows these proteins to combine with luminal IRE1*α*, then moderating excessive IRE1*α* activity to protect against cell injuries induced by maladaptive UPR (Zhang et al., 2019a). PDI is a kind of oxidoreductase which can catalyze disulfide bonds and it is the correct sequence of cysteine residue pairs with different redox potentials and substrate specificity (Ellgaard and Ruddock, 2005). Cysteine residues within the active site of PDI provide two electrons to assist disulfide bond formation, which results in reduction of the active site of PDI and oxidation of the substrate (Fu et al., 2020). The formation of disulfide bond in the ER is catalyzed by ERO1 (endoplasmic reticulum oxidoreductin 1) family of sulfhydryl oxidases. ERO1 oxidizes PDI and introduces disulfides into ER client proteins. ERO1 can couple disulfide transfer to PDI with reduction of molecular oxygen, forming hydrogen peroxide. Hence, ERO1 activity is a underlying source of ER-derived oxidative stress (Tavender and Bulleid, 2010). Research has found that ROS inhibitor MnTMPyP can inhibit the increase of ROS in ER stress and delay the activation of UPR, suggesting that ROS is the upstream signal molecule that triggers ERS-mediated apoptosis pathway and initiates ER stress-mediated apoptosis (Xu et al., 2018).

UPR as a protective means at the cellular level reduces the accumulation of unfolded or misfolded proteins on the ER. In the early stage of ER stress, ER mainly restore the normal function of ER for survival through activation of UPR to protect cellular damage caused by ER stress, which is a pro-survival response (Senft and Ronai, 2015; Frakes and Dillin, 2017).

### 4.2 Reactive Oxygen Species and Endoplasmic Reticulum Ca^2+^

#### 4.2.1 Reactive Oxygen Species Regulates Endoplasmic Reticulum Ca^2+^ Induced Endoplasmic Reticulum Stress

The endoplasmic reticulum is an important calcium reservoir, which regulates ER function, membrane transport and internal homeostasis through maintaining the balance of Ca^2+^ concentration inside and outside the cell. As a messenger molecule, Ca^2+^ participates in cell proliferation, differentiation, movement, muscle contraction, hormone secretion, glycogen metabolism, neuronal excitability, and other physiological activities, and then regulates gene and protein expression, secretion, metabolism, and apoptosis (Frakes and Dillin, 2017). Current studies have shown that disruption of calcium homeostasis in the ER can initiate early apoptosis signals (Rizzuto et al., 2012; Orrenius et al., 2015; Marchi et al., 2018). A study revealed that sodium nitrite (anti-tumor drug) can induce ER stress-mediated apoptosis in human NB4 cells in acute leukemia, certified selenite-induced production of ROS is the upstream of the JNK/ATF2 axis, application of ROS inhibitors partially inhibited the intracellular Ca^2+^ increase caused by sodium nitrite, suggesting that ROS is involved in the sodium nitrite-induced ERS-mediated apoptosis (An et al., 2013). Aoyama elucidated that rotenone, a mitochondrial complex I inhibitor, can cause ER hyperplasia, ER membrane lipid oxidative damage, increase the permeability of ER, cause the increase of cytosol-free Ca^2+^ and then induce ER stress and apoptosis. After using the ER stress inhibitor SUN N8075, mitochondrial ROS production obviously reduced and Ca^2+^ elevation induced by rotenone was significantly weakened, indicating the increase of intracellular free Ca^2+^ induced by rotenone is stimulated by ROS (Aoyama et al., 2021). Although some specific mechanisms are still being studied, ROS production and the increase of ER Ca^2+^ level are both crucial events in ER stress-mediated apoptosis.

#### 4.2.2 Reactive Oxygen Species Regulates IP3R and RyR Mediated Endoplasmic Reticulum Stress

At resting state, the concentration of Ca^2+^ in the ER is higher than that in the cytoplasm. There are three channels related with Ca^2+^ uptake and release for maintaining Ca^2+^ homeostasis on the ER: 1. Ca^2+^-release channels: RyRs (ryanodine receptors) and IP3Rs; 2. Ca^2+^ -ATPase or SERCA (sarcoplasmic-endoplasmic-reticulum Ca^2+^-ATPase) in transporting Ca^2+^ from cytoplasm to ER; 3. Ca^2+^-binding protein in ER cavity (such as calnexin and calreticulin) (Ong and Ambudkar, 2020). Studies have confirmed that the IP3R and RyR distributed on the sarcoplasmic reticulum can be regulated by the redox response (Ong and Ambudkar, 2020). ER includes the NOX subtypes of NADPH oxidase and co-exists with calnexin and calreticulin. The NOX subtype contains the main catalytic subunit GP91Phox (NOX2), which produces ROS mainly involved in cell signaling transduction. Recent studies suggest that NOX1 and NOX4 (NADPH Oxidase 4) can act on PDI, that is, ROS outside the mitochondria may also regulate ER function. Moreover, research has demonstrated NOX4 can act as a proximal signaling intermediate to transduce ER stress-related conditions to the UPR, and sulfonate and modify it through interaction with IRE1 to cause ER stress even cell injuries (Kim et al., 2016; Ochoa et al., 2018; Lee et al., 2020).

##### 4.2.2.1 Reactive Oxygen Species Regulates IP3-Mediated Ca^2+^ Release on Endoplasmic Reticulum

In the phosphatidylinositol signaling pathway, extracellular signaling molecules bind to the G-protein-coupled receptor on the cell surface to activate phospholipase C on the plasma membrane and hydrolyze PIP2 (4, 5-diphosphate phosphatidylinositol) into 2 s messengers, IP3 (1, 4, 5-triphosphate inositol) and DG (diacylglycerol). Extracellular signals are converted into intracellular signals, and this signaling system is also known as the double messenger system (Kankanamge et al., 2021). IP3R is a widely expressed channel for Ca^2+^ stores. IP3R can control the release of Ca^2+^ from stores into cytoplasm once being activated by IP3 and Ca^2+^ signaling at a lower concentration, and finally trigger downstream events. The closure of the IP3R channel caused by a rise in intracellular Ca^2+^ signals and the activation of the Ca^2+^ pump corporately restores the calcium store to a normal level (Zhang et al., 2020). Activation of NADPH oxidase in ER can improve the sensitivity of ER to IP3. NADPH oxidase-originated ROS can directly act on IP3R distributed on ER, causing its conformational changes, result in increased cytosolic free Ca^2+^ concentration, initiate intracellular Ca^2+^ signaling system, and regulate biological effects through Ca^2+^ dependent protein kinase II activity (Hu et al., 2000; Sakurada et al., 2019).

##### 4.2.2.2 Reactive Oxygen Species Regulates RyR—Mediated Ca^2+^ Release on Endoplasmic Reticulum

RyR is one of the intracellular Ca^2+^ release channels in the sarcoplasmic reticulum of muscle cells and endoplasmic reticulum of other cells and is the largest membrane protein molecule ever known. RyR in mammals can be divided into three subtypes: RyR1 (skeletal muscle type), RyR2 (myocardial type) and RyR3 (brain type). The expression level of RyR1 was highest in skeletal muscle, and RyR2 was mainly expressed in cardiomyocytes (Petrou et al., 2017) and some brain tissues. In cardiomyocytes, Ca^2+^ release of sarcoplasmic reticulum RyR2 is activated by influx of Ca^2+^ release through L-shaped calcium channels in the cell membrane, known as calcium-induced calcium release. RyR3 is widely distributed and expressed in many tissues. It is also activated by calcium-induced calcium release (Berridge, 2016). The increase of intracellular partial redox potential is the trigger point for important cellular functions. Studies found that RyR in the sarcoplasmic reticulum is sensitive to local redox potential, and mild oxidative stress can slightly increase intracellular redox potential, significantly enhance RyR activity and sensitize Ca^2+^ release mechanism, suggesting that ROS bidirectional regulation of ER Ca^2+^ may be concentration-dependent (Santulli et al., 2017; Kushnir et al., 2018). ROS promotes the synthesis of cADPR (cyclic adenosine diphosphate ribose) at low concentrations and opens RyR together with calmodulin. On the contrary, ROS leads to the oxidation of sulfhydryl groups of RyR and sarcoplasmic reticulum-related proteins into disulfide bonds, damaging Ca^2+^ release mechanisms (Avila et al., 2019; Kobayashi et al., 2021).

#### 4.2.3 Reactive Oxygen Species Regulates Endoplasmic Reticulum Ca^2+^ Uptake

In resting conditions, Ca^2+^ is absorbed by the cytoplasm into the ER cavity through SERCA pump and released into the cytoplasm by RyR and IP3R channels. Most of the Ca^2+^ released into the cytoplasm will be recaptured by the Ca^2+^ pump to maintain the Ca^2+^ concentration in the ER. Therefore, Ca^2+^ accumulation in ER is mainly accomplished through the activity of SERCA pump (Ivanova et al., 2020). Ca^2+^-ATPase, as a known promoter of ER stress, since oxidative damage suppresses Ca^2+^-ATPase activity, ROS can affect Ca^2+^ storage within ER by inhibiting Ca^2+^-ATPase, and hence affecting intracellular Ca^2+^ homeostasis and causing ER stress (Krylatov et al., 2018). Studies have revealed ROS production and ER Ca^2+^ pump were sensitive to oxidative damage in ischemia and hypoxia cell models. ROS can make proteins in ER cavity be oxidized and retained, thus inhibiting protein synthesis, and leading to cell apoptosis. Puteney et al. found that Ca^2+^ releasing from the ER *via* IP3R causes decreased ER calcium reserves, Ca^2+^ can enter the cell from the extracellular, a process known as SOCE (store-operated Ca^2+^ entry) (Putney, 1986) Ivan demonstrated that exposure to environmental or cell-intrinsic ROS can affect Ca^2+^ homeostasis, modify multiple pathways and cause cell injuries (Bogeski et al., 2012). ROS production can induce Ca^2+^ release within the ER to cause ER stress, whereas Ca^2+^ release within the ER further stimulates Ca^2+^ aggregation on the mitochondria, causing mitochondrial damage and subsequently inducing apoptosis. As illustrated above, calreticulin is the main Ca^2+^ binding chaperone in the ER lumen, which binds to Ca^2+^ through the C-domain in a high capacity and increases the Ca^2+^ storage capacity of the ER lumen (Venkatesan et al., 2021). Leta found that calreticulin acts directly with SERCA2b glycosylated C-terminal tails to regulate Ca^2+^ storage in ER. When ER Ca^2+^ is sufficient, the activity of ATP-ase can be inhibited and intracellular Ca^2+^ transport to ER stops (Johnson et al., 2014).

Elevated ROS levels send feedback signals that stimulate ER stress. ER stress promotes the correct folding of the wrong proteins and degrades the proteins that cannot be folded. Transient ER stress helps to restore cell homeostasis, but long-term and high-intensity ERS can lead to multiple pulmonary diseases.

## 5 Endoplasmic Reticulum Stress in Pulmonary Disease

**FIGURE 2:**
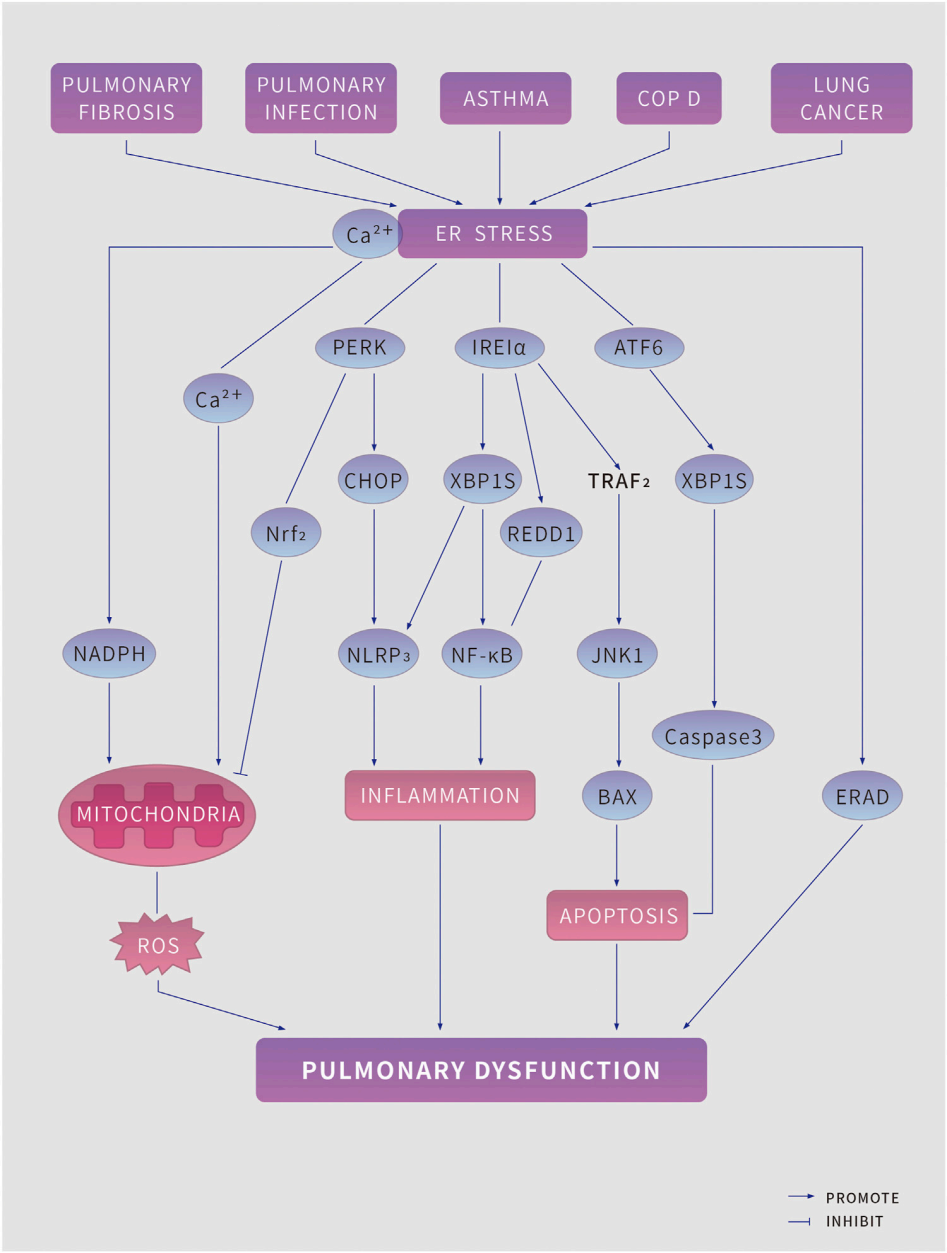
ER stress in pulmonary disease. Pulmonary pathologies, including pulmonary fibrosis, pulmonary infection, asthma, Chronic obstructive pulmonary disease and lung cancer lead to endoplasmic reticulum (ER) stress and induce the unfolded protein response (UPR) (which has three branches, mediated by protein kinase RNA-like ER kinase (PERK), Inositol-requiring protein 1*α* (IRE1*α*) and activating factor 6 (ATF6) to tackle ER stress. However, if the UPR fails to restore ER homeostasis, it might induce risk factors for pulmonary disease, including increased reactive oxygen species (ROS) production, inflammation, and apoptosis, which further aggravate pulmonary disease. BAX, BCL-2-associated X protein; CHOP, C/EBP homologous protein; ERAD, ER-associated degradation; JNK1, JUN N-terminal kinase 1; The NK-kappa B, nuclear factor kappa B predominate; NLRP3, NACHT, LRR and PYD domains-containing protein 3; NADPH, nicotinamide adenine dinucleotide phosphate; NRF2, nuclear factor erythroid 2-related factor 2; REDD1, protein regulated in development and DNA damage response 1; TRAF2, TNF receptor associated factor 2; XBP1s, spliced X-box-binding protein 1.

As ER stress aggravates, persistent UPR triggers programmed cell death. The occurrence and development of a variety of lung diseases are closely related to intracellular ROS, ER stress and UPR. (Figure 2)

### 5.1 Pulmonary Fibrosis

Pulmonary fibrosis is a kind of lung diseases characterized by inflammatory damage and tissue structure destruction. Pulmonary fibrosis is usually caused by excessive normal tissue repair resulting in vast proliferation of fibroblasts and abnormal accumulation of extracellular matrix. Cells activate UPR signaling to maintain ER homeostasis. Although the short-time UPR is beneficial to maintaining the cellular protein balance, it can cause lung cell dysfunction or even apoptosis when the UPR is stimulation is overwhelmed (Noble et al., 2012; Hetz et al., 2020).

#### 5.1.1 Idiopathic Fibrosis of the Lung

IPF (idiopathic fibrosis of the lung) is a serious interstitial lung disease that can cause progressive loss of lung function, and its pathological mechanism is constantly being updated. Many cells in the lung parenchyma, including alveolar epithelial cells, macrophages, and fibroblasts, are affected by UPR during the pathological process of IPF. CE (Alveolar epithelial cells) are mainly divided into AEC I (alveolar epithelial cells I), and AEC II (alveolar epithelial cells II). AEC II cells are the progenitor cells required to maintain alveolar integrity. Loss of AEC II function and apoptosis are considered as important processes in the initial stage and development of IPF (Hetz et al., 2020). Researchers took samples of peripheral transplanted lung tissues, analyzed through protein detection, and found that AEC II in IPF can produce much UPR related proteins such as ATF6, ATF4 and CHOP. On the contrary, it does not exist in COPD and normal organ donors, indicating that in IPF patients, UPR activation can lead to the apoptosis of AEC II cells in the inner surface of the fibrotic site and be replaced by fibrous tissue (Korfei et al., 2008; Lawson et al., 2011). Timothy and his colleagues used tunicamycin to induce UPR activation, which enhanced AEC death and pulmonary fibrosis in IPF mice models, demonstrating UPR activation may increase AEC vulnerability, lead to enhanced lung injuries and IPF occurrence. Although UPR cannot directly aggravate the occurrence of IPF, it can improve the incidence probability of IPF in patients (Kropski et al., 2012). In many IPF patients, herpesviruses have been found to be closely associated with the occurrence of IPF. Herpesviruses are frequently found in the IPF lungs and can induce the UPR. Modification and maturation of viral glycosylated envelope proteins during virus replication may induce ER stress, while ER stress play double-acting roles in virus infection. Herpesvirus infection can directly promote UPR activation, causing decreased immune function induced malignant pulmonary fibrosis and IPF (Lee et al., 2009; Tanjore et al., 2012; Liu et al., 2021).

#### 5.1.2 Cystic Fibrosis

Pulmonary cystic fibrosis (CPF or CF) is a congenital disease with familial autosomal recessive inheritance. It is most common among white North Americans and rarely seen in other races. As an exocrine gland lesion, the gastrointestinal tract and respiratory tract are often affected (Elborn, 2016). The airway of patients with CF presents a persistent and intense inflammatory response, and AMs (alveolar macrophage) play key roles in the pathogenesis of CF (Hey et al., 2021). Some AMs can secrete many inflammatory mediators such as TNF-*α* and IL-6 induction (Lambertsen et al., 2012). XBP1 cut by IRE1 pathway mRNA in UPR affects peripheral macrophages and produces inflammatory effects (Lubamba et al., 2015). It was found that the UPR activated the expression of XBP1 in AMs in CF patients, and lipopolysaccharide was associated with the increased level of transcription factor XBP-1 in the UPR. Knockdown of XBP-1 in cultured dTHP-1 (differentiated human acute monocytic leukemia-1) cells reduced the inflammatory response of CF patients. Inflammatory factors in CF patients can be regulated by the expression of XBP-1 in UPR, and AMs in CF patients can promote the occurrence of airway inflammation in CF patients by upregulating xBP-1-mediated cytokines (Zhu et al., 2012; Lubamba et al., 2015). CFTR (cystic fibrosis transmembrane conductance regulator) is a central regulator of chlorine channel and epithelial function. Loss of CFTR function in airway can lead to mucus thickening, reduced mucosal ciliary clearance, chronic infection, and respiratory failure. UPR can induce the binding of ATF6 to the minimum promoter of CFTR, leading to the inhibition of CFTR transcription and reduce expression of CFTR (Trouvé et al., 2021).

### 5.2 Pulmonary Infections

Pulmonary infection refers to the infectious disease of pulmonary parenchyma and interstitial tissues, mainly caused by virus, bacteria, or other microbial infection, mainly in the form of pneumonia in clinical. UPR signaling play important roles in the secretion of inflammatory factors. Since inflammatory cells tend to have large secretory requirements and are therefore particularly dependent on a well-developed and abundant ER, the presence of infection-induced stimuli may induce an intracellular UPR response. Inflammatory factors such as TNF- (tumor necrosis factor), IL-1 (interleukin-1), and IL-6 can also cause the activation of three UPR pathways in cells, thus causing the acute phase response of lung inflammation (Grootjans et al., 2016). UPR activation and infection are mutually reinforcing and antagonizing each other (Bettigole and Glimcher, 2015).

#### 5.2.1 Acute Pneumonia

There are many causes of the acute pneumonia response, where acute lung injury is the most important cause. In acute pneumonia caused by acute lung injury, the UPR activation plays an important role in cellular autophagy, as well as in apoptosis (Bettigole and Glimcher, 2015). In lipopolysaccharide-induced acute lung injury mice model and human alveolar epithelial cell model, the UPR inhibitor 4-PBA (4-phenylbutyric acid) blocks the activation of NF-κB pathway and reduces the release of pro-inflammatory mediators including TNF-*α*, IL-1*β* and IL-6. 4-PBA reduces the activation of inflammation by inhibiting UPR pathway and the mechanism is likely to be related to PERK-eIF2*α*-mediated translation reduction promoting NF-κB activation, suggesting that UPR is a crucial promoter of lipopolysaccharide-induced inflammation. 4-PBA leads to decreased autophagy, which plays a protective role in acute pneumonia through the classical AKT/mTOR signaling pathway and exacerbates lipopolysaccharide-induced A549 cytotoxicity through the 3-MA (autophagy inhibitor 3-methyladenine). 4-PBA inhibits UPR activation and has protective effects on autophagy in acute pneumonia models. UPR can stimulate the activation of NF-κB pathway, leading to the translocation of free NF-κB into the nucleus, possibly through conformational changes in cytoplasmic regions induced by IRE1*α* autophosphorylation. The IRE*α* -TRAF2 complex binds to the adaptor protein TNF-*α* receptor-associated factor 2 to recruit IκB kinase and phosphorylates IκB, resulting in degradation of IκB and nuclear NF-κB translocation (Urano et al., 2000). Jiang et al. investigated whether ER stress during simulated microgravity induced endothelial inflammation and apoptosis in HUVECs (human umbilical vein endothelial cells), and they found that the increase of apoptosis in HUVECs during clinorotation was significantly suppressed by inhibiting ER stress, iNOS activity, NF-κB/IκB, and the NLRP3 inflammasome signaling pathway, indicating ER stress-dependent activation of iNOS/NO-NF-κB signaling and NLRP3 inflammasome contributes to chronic injury endothelial inflammation and apoptosis associated with microgravity (Jiang et al., 2020).

#### 5.2.2 Allergic Bronchopulmonary Aspergillosis

Aspergillus fumigatus is an opportunistic pathogen that is responsible for a life-threatening fungal infection called as invasive aspergillosis. The sensitization of AF (aspergillus fumigatus) was associated with severe allergic pulmonary inflammation, and allergic bronchopulmonary aspergillosis (ABPA) was the most common disease in allergic mycosis (Krishnan and Askew, 2014). Studies found that in clinical studies, glucoregulatory protein GRP78 was increased in lung tissues of patients with allergic bronchopulmonary aspergillus. Lee found that utilizing of the ER stress inhibitor or a ROS scavenger improved AF-induced allergic inflammation. The PI3K-*δ* inhibitor reduced AF-induced mitochondrial ROS generation and ER stress accordingly ameliorated. They revealed that PI3K-*δ* regulates AF-induced steroid-resistant eosinophilic allergic lung inflammation through ER stress (Lee et al., 2016). Wang revealed the effects and mechanisms of resveratrol on fungus-induced allergic airway inflammation, and they demonstrated resveratrol alleviated the AF-exposed allergic inflammation and apoptosis through inhibiting ER stress *via* Akt/mTOR pathway, exerting therapeutic effects on the fungus-induced allergic lung disorder (Wang et al., 2022). These studies revealed the UPR is a critical step in ABPA production.

### 5.3 Asthma

The bronchial epithelium contains a variety of cell types with high protein synthesis and secretion, which are prone to ER stress (Schögler et al., 2019). In bronchial asthma, the existence of airway inflammation state and other factors are considered to induce UPR, and they are mutually causal and interactive with airway inflammation. Current studies suggest that ER stress is closely related with the dysregulation of Ca^2+^ homeostasis in airway epithelial cells, abnormal secretion of hyaluronic acid and mucin, recruitment of inflammatory cells by cytokines, and abnormal immunomodulatory state in the pathogenesis of bronchial asthma (Russell and Brightling, 2017). The expression of UPR marker protein GRP78 was significantly increased in peripheral blood monocytes and bronchoalveolar lavage fluid of asthmatic mice models, suggesting that the UPR pathway may cause the development of asthma by activating NF-κB, indicating UPR can also stimulate the activity of immune, and get involved in immune response (Kim et al., 2013b; Kim and Lee, 2015). The CHOP protein (also known as GADD153 or DDIT-3) is an important signal molecule in ER stress, and its specific mechanism in asthma has been gradually studied. It has been found that T cells treated with curcumin can induce activation of the PERK and IRE1 pathways in UPR and induce upregulation of stimulating transcription factors XBP-1 and CHOP in CD4^+^ lymphocytes and T lymphocytes, ultimately leading to apoptosis of T cells (Zhang et al., 2012; Lin et al., 2019). ORMDL3 (Orosomucoid like 3) has been considered as significant regulator of asthma in genetic association studies, David and his colleagues found transfection of ORMDL3s in human bronchial epithelial cells *in vitro* induced expression of metalloproteases (MMP-9), CC chemokines (CCL-20), CXC chemokines (IL-8, CXC-10, CXCL-11) and selectively activated ATF6 and SERCA2B. They furtherly revealed ORMDL3 may be associated with ER-UPR pathway in asthma. It was reported that the expression of ORMDL3 increased UPR response, and related genes signaling pathways of UPR were significantly upregulated. Ca^2+^ released by ER calcium influx decreased in ORMDL3 knockout models, and UPR was correspondingly weakened. The increased expression of ORMDL3 gene in asthmatic patients is probably caused by regulating UPR pathway affecting Ca^2+^ concentration (Cantero-Recasens et al., 2010).

### 5.4 Chronic Obstructive Pulmonary Disease

COPD (chronic obstructive pulmonary disease) is a kind of pulmonary disease characterized by persistent respiratory symptoms and airflow restriction, which is not completely reversible and develops progressively, mainly affecting the lungs (Labaki and Rosenberg, 2020). COPD is often associated with smoking, and smoking exposure damages the proteasome itself, resulting in massive accumulation of insoluble proteins in airway epithelial cells, alveolar epithelial cells, and macrophages, ultimately causing UPR. COPD patients have more mucus cells in their respiratory tract, often secreting a lot of mucus. IRE1*β* (inositol-requiring enzyme 1beta) of airway goblet cells plays an important role in mucin cell phenotype and secretion of mucin5AC and mucin5B (Cloots et al., 2021). Michael found that in HEK293 cells models with purified protein, IRE1*α* is closely related with IRE1*β* diminishing expression and inhibiting signaling. IRE1*β* can assemble with and inhibit IRE1*α* to suppress stress induced XBP1 splicing to affect the UPR (Grey et al., 2020). Research have shown that hypoxic stimulation can activate the dissociation of molecular chaperone GRP78 and activate UPR to repair protease and autophagy functions of cells. Studies also found that the expression of UPR signaling molecules including GRP78, XBP1, CHOP and other proteins were not found in every COPD patient, suggesting that the UPR pathway was activated only in a part of COPD population (Min et al., 2011; van 't Wout et al., 2015). Mitochondrial oxidative stress is specifically significant in COPD, and Nrf2 (nuclear factor-E2-related factor 2) is a major regulator of antioxidant response element-driven cytoprotective protein expression. ER stress-mediated UPR and insufficiency of antioxidant Nrf2 has been implicated in cigarette smoking-induced COPD, suggesting the potential of Nrf2 in the treatment of COPD (Fratta Pasini et al., 2016; Liu et al., 2019).

### 5.5 Lung Cancer

Lung cancer is the largest tumor threatening human beings (Nasim et al., 2019). Lung cancer cells often tackle ischemia, hypoxia, metabolism, and other changes. To cope with these extreme factors, the cells themselves also produce corresponding changes, resulting in the activation of UPR pathway. The role of ER stress and the UPR is well demonstrated in a lung cancer (Dastghaib et al., 2021).

#### 5.5.1 Lung Adenocarcinoma

The most common type of lung cancer is adenocarcinoma, comprising around 40% of all lung cancer cases, and lung adenocarcinoma is still one of the most aggressive and rapidly fatal tumor types with overall survival less than 5 years (Denisenko et al., 2018). Many studies have shown that adenocarcinoma is often accompanied by activation of the UPR pathway. Lu found that ER stress can induce the high expression of the transcription factor XBP1 in 2D and 3D culture lung adenocarcinoma cells and promote the proliferation of lung adenocarcinoma in 3D culture *in vitro*. Knockdown the expression of XBP1 can block cell growth induced by Tm/Tg treatment. Meanwhile, LOX gene, as a key downstream sensor of XBP1, can block XBP1-induced cell proliferation by knocking down the expression of LOX, suggesting that XBP1 can regulate the proliferation of lung adenocarcinoma cells through LOX (Yang et al., 2017). It was found that asterosaponin 1 increased ER dilation and cytosolic Ca^2+^ concentration, enhanced the expression of GRP78 and GRP94 in a dose-and-time-dependent manner and increased the expression of CHOP, Caspase-4 and JNK, the three UPR related apoptotic molecules. Finally, the proliferation of A549 cells was inhibited and apoptosis was promoted (Zhao et al., 2011).

#### 5.5.2 Large Cell Carcinoma

Large cell carcinoma is a barely descriptive term indicating a subtype of lung cancer with no specific features of small-cell lung cancer, adenocarcinoma or squamous cell carcinoma (Weissferdt, 2014). Azam et al. have found that GRP78 is closely related to the regulation of large cell carcinoma. The expression of GRP78 in tumor of patients with large cell carcinoma is more than three times that of the negative control. Both miR-495 and miR-199a-5p can promote the malignant transformation of tumor cells. Increased GRP78 in cells can downregulate Mir-495 and Mir-199a-5p in NSCLC, and overexpression or inhibition of miR-495 and miR-199a-5p can also significantly change the expression of GRP78 and XBP1 level. UPR and microRNA interact to promote the occurrence of cancer (Ahmadi et al., 2017). Pau and his colleagues found that the anti-cancer molecule ABTL0812 increases cellular long-chain dihydroceramides by impairing DEGS1 (delta 4-desaturase, sphingolipid 1) activity, which resulted in persistent ER stress and activated UPR *via* ATF4-DDIT3-TRIB3 that ultimately promotes cytotoxic autophagy in cancer cells (Muñoz-Guardiola et al., 2021). Studies have proved that in large-cell lung carcinoma H460 treated with cisplatin, the cell viability is distinctly reduced after adding ERS inhibitor 4-PBA (4-phenylbutyric acid). Intracellular caspase-3 gene and cytochrome C were significantly upregulated and promoted cell apoptosis, confirming that chemotherapy drugs can activate UPR and enhance drug resistance of tumor cells (Shi et al., 2016). Blocking the adaptive pathway of ER stress or promoting the apoptotic pathway could be a promising anti-cancer strategy.

## 6 Potential Therapeutic Interventions in Pulmonary Disease

Given the significant characteristic in the pathogenesis of pulmonary disease, strategies to target ER proteostasis, UPR signaling, and ROS are crucial for disease intervention. We discuss strategies as following.

### 6.1 Pharmacotherapy

#### 6.1.1 Chemical Chaperones

4-PBA. 4-phenyl butyric acid, a low-molecular-weight aromatic fatty acid, affect the activation of LPS-induced inflammation human alveolar epithelial cell in acute lung injury models. It further prevents the activation of the NF-κB pathway, decreases the release of the inflammatory mediators through regulating ER stress (Zeng et al., 2017).

TUDCA. TUDCA (Tauroursodeoxycholic) is a hydrophilic bile acid with chaperone properties that is produced endogenously in humans at very low levels. Tong found that the TUDCA can moderate pulmonary ER stress and epithelial-mesenchymal transition in bleomycin-induced lung fibrosis (Tong et al., 2021). TUDCA can inhibit CHOP expression and the bleomycin -induced pulmonary fibrosis and inflammation, suggesting TUDCA may be a promising strategy for preventing pulmonary fibrosis (Tanaka et al., 2015).

#### 6.1.2 Salubrinal

EIF2A phosphorylation levels are controlled by two phosphatase complexes, hydrophobic the protein Phosphatase 1 (PP1) catalytic subunit complexed with either constitutively expressed PPP1R15B (PP1 regulatory subunit 15B) or the ER Stress-inducible isoform PPP1R15A, which is an ATF4 -- CHOP Target (PP1). Salubrinal is a cell-permeable, small-molecule inhibitor of the PP1 complex (Liu et al., 2014). Wang found that Salubrinal attenuated A549 cells from Paraquat-induced damages through upregulating the PERK-eIF2*α* signaling (Wang et al., 2018b). Salubrinal may be a promising therapeutic strategy to suppress ER stress in pulmonary dysfunctions.

#### 6.1.3 Metformin

Metformin is an antidiabetic drug that has been proved to be beneficial to other diseases independent of its glycaemia effects. Francesca found that metformin may ameliorate cigarette smoke-induced pathologies of emphysematous COPD through the AMPK (AMP-activated protein kinase) pathway (Polverino et al., 2021).

#### 6.1.4 m-TOR Inhibitor

Qin demonstrated ER stress-induced cell death was mediated by autophagy which was partly attributed to the inactivation of the mTOR (mammalian target of rapamycin) (Qin et al., 2010). Tian found that ginkgo biloba leaf extract attenuates atherosclerosis in streptozotocin-induced diabetic ApoE^−/−^ mice by Inhibiting ER stress *via* restoration of autophagy through the mTOR Signaling Pathway (Tian et al., 2019), which inspired us that the mTOR pathway is expected to become a promising target of ER stress-induced lung diseases.

#### 6.1.5 Alanyl Beta Muricholic Acid

A*β*M acid can resolve the inflammatory and UPR in inflammatory diseases (Vander Zanden et al., 2019). Emily proved that A*β*M acid-based therapy can inhibit the allergen-induced UPR and allergic airway disease in mice *via* preferential binding of ATF6*α*, suggesting a novel avenue to treat allergic asthma using A*β*M (Nakada et al., 2019).

#### 5.1.6 N*-*(2-Hydroxy-5-Methylphenyl)-3-Phenylpropanamide (147)

Pharmacologic arm selective UPR signaling pathway activation is emerging as a promising strategy to improve imbalances in ER proteostasis implicated in diverse diseases. The compound 147 was considered to activate ATF6-regulated signaling associated with localized metabolic activation and selective covalent modification of ER stress (Paxman et al., 2018). Therefore, the compound 147 is a promising strategy in preventing ER stress-induced pulmonary disease.

#### 6.1.7 Melatonin

Melatonin is an anti-inflammatory molecule, which has been effective in acute lung injury induced by many conditions (Tordjman et al., 2017). Zhao revealed that melatonin obviously inhibited the activation of NLRP3 inflammation, ER stress and epithelial-mesenchymal transition during bleomycin-mediated pulmonary fibrosis in mice models (Paxman et al., 2018).

#### 6.1.8 Ursolic Acid

Li found that ursolic acid derivative has preventive effects on particulate matter 2.5-induced COPD, causes obvious suppression in PM2.5-induced increase in oxidative stress markers and inflammatory cytokines, suggesting ursolic acid derivative can be a promising therapeutic treatment for PM2.5-induced COPD (Li et al., 2020).

#### 6.1.9 Resveratrol

Resveratrol, a natural polyphenol, is beneficial in supporting its numerous biological properties including antioxidant, anti-inflammatory, ant obesity, antidiabetic, and ant-ischemic activities. The compound also has beneficial effects on cognitive function and lung health (Ahmadi et al., 2021). Wang demonstrated that resveratrol attenuates inflammation and apoptosis through alleviating ER stress *via* Akt/mTOR pathway in fungus-induced allergic airways inflammation, providing therapeutic strategies for fungus-induced allergic lung disease (Wang et al., 2022).

### 6.2 Strategies Target Reactive Oxygen Species

Diets poor in antioxidants are related with lung dysfunction and a risk factor for the development of COPD. Dietaries include Vitamin C (ascorbic acid), Vitamin E (*α*-tocopherol), resveratrol, and flavonoids has always been studied in improve lung function or clinical features of COPD (Rana, 2020). Andras implied ascorbate may be involved in oxidative protein folding and creates a link between the disulfide bond formation (oxidative protein folding) and hydroxylation, providing promising strategies in preventing and treating pulmonary disease (Szarka and Lőrincz, 2014).

NRF2-mediated heme oxygenase was upregulated by UPR activation in PC12 cells. and GCLC (glutamate cysteine ligase catalytic) subunit expression increases. Heme oxygenase and GCLC can lead to COPD disease aggravation, indicating increasing Nrf2 activity as a treatment strategy in COPD (Digaleh et al., 2013; Niforou et al., 2014; Wu et al., 2015).

### 6.3 Lifestyle Adjustments

Lifestyle adjustments including physical exercise, smoking cessation and diet are a novel approach to modulate ER stress to protect against pulmonary disease (Itoh et al., 2013; Pedersen and Saltin, 2015; Schuller, 2019).

ROS-ER stress-associated therapy is becoming recognized as a crucial treatment strategy for pulmonary disease. Efficiently targeting ER stress in an organelle-specific or organ-specific manner holds promise for preventing and treating pulmonary disease.

## 6 Conclusion

Massive attention has been paid to the role of ROS and ER stress in the pathogenesis of pulmonary disease over the past years. The ER coordinates secretory and transmembrane proteins folding and translocation, controls cellular calcium uptake, storage and signaling.

The ER stress promotes the correct folding of faulty proteins and the degradation of misfolding proteins. Transient ER stress contributes to the homeostasis of cells, while sustained and overwhelmed ER stress can lead to several lung diseases. Unfold protein response is the most important reaction in ER stress. UPR and calcium initial signaling are key mechanisms of ER stress-mediated apoptosis. We summarized the mechanisms of ER stress, key regulators, related pathways, and associated lung diseases, including pulmonary fibrosis, pulmonary infections, asthma, COPD, and lung cancers. We also provided latest interventions target ER stress and ROS in pulmonary disease.

ER homeostasis and ER stress, especially the biological entities of ER stress, have been extensively described in many reports. However, there are still many unanswered questions. Although some protein-regulatory promoter compounds targeting ER stress or UPR components have shown potential in preventing and treating lung disease, clinical trials evaluating these agents targeting ER stress or UPR are still lacking. ROS serves as an upstream signal of ER stress, and the study of their internal relationship and mechanism of action need us further understand and provide new ideas for the development of drugs for pulmonary diseases, and further research is needed to provide a new approach for tumor treatment.
